# Novel strategies for the characterization of cancellous bone morphology: Virtual isolation and analysis

**DOI:** 10.1002/ajpa.24272

**Published:** 2021-04-03

**Authors:** Alessio Veneziano, Marine Cazenave, Fabio Alfieri, Daniele Panetta, Damiano Marchi

**Affiliations:** ^1^ Synchrotron Radiation for Medical Physics (SYRMEP) Elettra‐Sincrotrone Trieste S.C.p.A Trieste Italy; ^2^ Skeletal Biology Research Centre at the School of Anthropology and Conservation University of Kent Canterbury UK; ^3^ Department of Anatomy and Histology Sefako Makgatho Health Sciences University Pretoria South Africa; ^4^ Institut für Biologie Humboldt Universität zu Berlin Berlin Germany; ^5^ Museum für Naturkunde Leibniz‐Institut für Evolutions‐ und Biodiversitätsforschung Berlin Germany; ^6^ Istituto di Fisiologia Clinica Consiglio Nazionale delle Ricerche (CNR) Pisa Italy; ^7^ Department of Biology Università di Pisa Pisa Italy; ^8^ Evolutionary Studies Institute and Centre for Excellence in PalaeoSciences University of the Witwatersrand Johannesburg South Africa

**Keywords:** bone complexity, bone segmentation, primates, skeletonization, trabecular architecture

## Abstract

**Objectives:**

The advent of micro‐computed tomography (μCT) made cancellous bone more accessible than ever before. Nevertheless, the characterization of cancellous bone is made difficult by its inherent complexity and the difficulties in defining homology across datasets. Here we propose novel virtual methodological approaches to overcome those issues and complement existing methods.

**Materials and methods:**

We present a protocol for the isolation of the whole cancellous region within a μCT scanned bone. This method overcomes the subsampling issues and allows studying cancellous bone as a single unit. We test the protocol on a set of primate bones. In addition, we describe a set of morphological indices calculated on the topological skeleton of the cancellous bone: node density, node connectivity, trabecular angle, trabecular tortuosity, and fractal dimension. The usage of the indices is shown on a small comparative sample of primate femoral heads.

**Results:**

The isolation protocol proves reliable in isolating cancellous structures from several different bones, regardless of their shape. The indices seem to detect some functional differences, although further testing on comparative samples is needed to clarify their potential for the study of cancellous architecture.

**Conclusions:**

The approaches presented overcome some of the difficulties of trabecular bone studies. The methods presented here represent an alternative or supporting method to the existing tools available to address the biomechanics of cancellous bone.

## Graphical abstract

The study of cancellous bone is hindered by its inherent complexity and difficulties in finding homology across samples. We introduce novel methods to overcome these issues. First, we propose a protocol to isolate cancellous bone from μCT scanned bones. Second, we developed an approach based on skeletonization to quantify cancellous complexity.

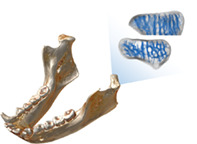

## 1 INTRODUCTION

Cancellous bone responds to variations in nature, direction, frequency and magnitude of load throughout life (Carter et al., 1989; Goldstein et al., 1991; Huiskes et al., 2000; Kivell, 2016; Macchiarelli et al., 1999; Rafferty & Ruff, 1994), as well as to nutrient intake (Chen et al., 2002; Gunnes & Lehmann, 1995, 1996; Tu et al., 2017) and hormones (Andreassen & Oxlund, 2001; Miyakoshi, 2004). Therefore, research has turned to cancellous bone to address issues of bone usage, biomechanics and stress in forensic (e.g., Villa et al., 2013) and biological anthropology (e.g., Cazenave et al., 2019; Georgiou et al., 2019; Macchiarelli et al., 1999; Rafferty & Ruff, 1994; Ryan & Shaw, 2012; Scherf et al., 2013; Tsegai et al., 2018a), archaeology (e.g., Bishop et al., 2017; Kneissel et al., 1994; Scherf et al., 2016) and paleontology (e.g., Bishop et al., 2017, 2018; Sinclair et al., 2013). The advent of micro‐computed tomography (μCT) made bone internal structures noninvasively accessible (Fajardo et al., 2002) and allowed the virtual manipulation of cancellous bone (Odgaard, 1997), thus expanding the methodological opportunities.

Cancellous bone is composed of intertwined trabecular elements forming an intricate structure with no analogy to regular solid shapes and, therefore, hard to describe through traditional morphometrics (Hildebrand et al., 1999; Odgaard, 1997). Furthermore, the developing trabecular lattice is modeled throughout life by the complex interaction between endogenous and exogenous factors (Cooper, 1990; Little et al., 2011). Given the multifaceted nature of its development, the characterization of cancellous bone should consider complexity as a feature useful to understand its variability and function.

Previous work focused on subsamples of trabecular regions to analyze the cancellous bone (e.g., Fajardo & Müller, 2001; Moon et al., 2004; Räth et al., 2008; Ryan & Ketcham, 2002). These subsamples bear only local information (Fajardo & Müller, 2001; Georgiou et al., 2019; Kivell et al., 2011) and their position and orientation are difficult to define homologously, especially across species with different external bone morphology (Georgiou et al., 2019). Studies have also observed that some measurements on cancellous bone depend on the size of the subsample (Lazenby et al., 2011) and that cancellous properties are not homogeneous across the whole epiphysis (Sylvester & Terhune, 2017). Because of these limitations, an approach focusing on the whole cancellous region could provide additional functional information. Indeed, prior research has shown that extended cancellous areas within an epiphysis can lead to more meaningful statistical comparisons (Sylvester & Terhune, 2017). In addition, characterizing the cancellous properties across the whole epiphysis allows mapping the differences across the bone and articular surfaces. This approach has been successfully used in previous research (Georgiou et al., 2019; Sylvester & Terhune, 2017; Tsegai et al., 2013; Tsegai et al., 2018b).

In summary, the study of cancellous bone can be made difficult by (I) its inherent complexity and (II) the lack of clear homology between cancellous regions in interspecific datasets. In this work we address both issues. First, we present a reproducible protocol for isolating cancellous structures from stacks of μCT images, thus allowing the analysis of cancellous regions without subsampling. Second, we present a set of indices measured on the topology of the cancellous structure that quantify new aspects of cancellous complexity.

## 2 MATERIALS AND METHODS

**FIGURE 1:**
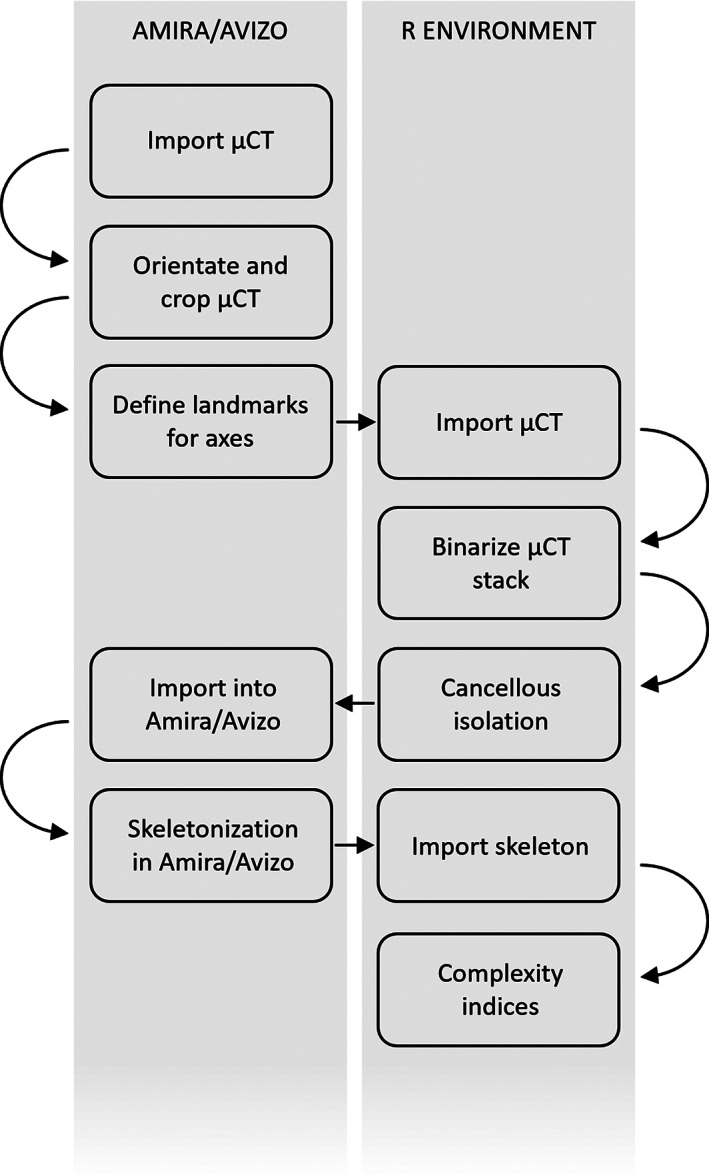
The workflow of the methodological approach introduced in this work. Actions are divided based on the environment where they are performed (R or Amira/Avizo)

Most of the operations used in this work are currently available in the R environment (R Core Team, 2020). The isolation of cancellous bone and the calculation of complexity indices are available in the package “indianaBones,” currently available on Github (github.com/AlessioVeneziano/indianaBones). A working example is provided in the Supplementary information. Following, we describe the general workflow making use of the R environment whenever possible but also highlighting the steps performed in the Amira/Avizo software (FEI Visualization). Figure 1 shows a diagram of the workflow.

### 2.1 Orientate, crop the μCT stack, and define axes

The μCT stack is cropped and orientated to facilitate further processing. This can be easily performed in software packages designed for handling image stacks. In Amira/Avizo, cropping can be performed in the *Volume editor* and orientation of the image stack is performed using the *obliqueSlice* function. Once cropped and orientated, the μCT stack can be exported in several file formats (e.g., TIFF stack, DICOM, NIfTI). For the later calculation of some of the complexity indices, measurements are needed to provide scaling factors or reference axes for orientation. These measurements and axes can be defined in any software for image stack processing by collecting landmarks. In Amira/Avizo this can be performed using the *Landmark* editor: the recorded landmarks can be then exported in ASCII format and imported into R using the *read.amira.set* function in the "Arothron" R package (Profico et al., 2020).

### 2.2 Import the μCT stack and binarize

In R, the μCT stack can be imported in several ways. TIFF images can be imported using the R package “tiff” (Urbanek, 2020) and the function *readTiffStack* available in the package “indianaBones” extends this to image stacks. DICOM and NIfTI stacks can be imported using the R package “oro.dicom” and “oro.nifti” (Whitcher et al., 2011), respectively. The stack is imported as a 3D array of dimensions K x M x N, where K and M are the image width and height in pixels, and N is the number of images in the stack. After importing, the binarization takes place, returning images with the bone and the background only (respectively as white and black pixels). This transformation is necessary because the protocol for the isolation of cancellous bone works on binary images only. Image binarization can be achieved via any suitable image segmentation method (Pham et al., 2000). One method widely used is the Otsu thresholding (Otsu, 1979; Vala & Baxi, 2013), which is available in the R package “EBImage” (Pau et al., 2010). The binarization can be performed in other software packages designed for image stack processing.

### 2.3 Isolation of the cancellous bone

To isolate the cancellous from the cortical bone, the binarized stack is processed using the function *splitBone* in the R package “indianaBones.” This function implements a protocol using a combination of the image processing operators “Dilation” and “Erosion” (Serra, 1982; Urbach & Wilkinson, 2007), which respectively enlarge and shrink features in binary images according to a pattern specified by a “structuring element.” Dilation and erosion are performed using the package “EBImage,” called by the “indianaBones” function *splitBone*. Structuring elements are matrices of odd dimensions that identify the pixel in the image being processed (Urbach & Wilkinson, 2007). Each pixel in the image is the center of the structuring element. The neighboring pixels are the cells of the element that surround its center. Each pixel is modified based on the value of its surrounding pixels, according to the pattern in the structuring element. In the case of dilation and erosion, each white pixel (region of interest) in the image will grow and shrink over its neighboring pixels in the fashion specified by the values in the structuring element. This process is illustrated in the supplementary information (Figure S1). Structuring elements can be defined using the *makeBrush* function in the “EBImage” R package.

**FIGURE 2:**
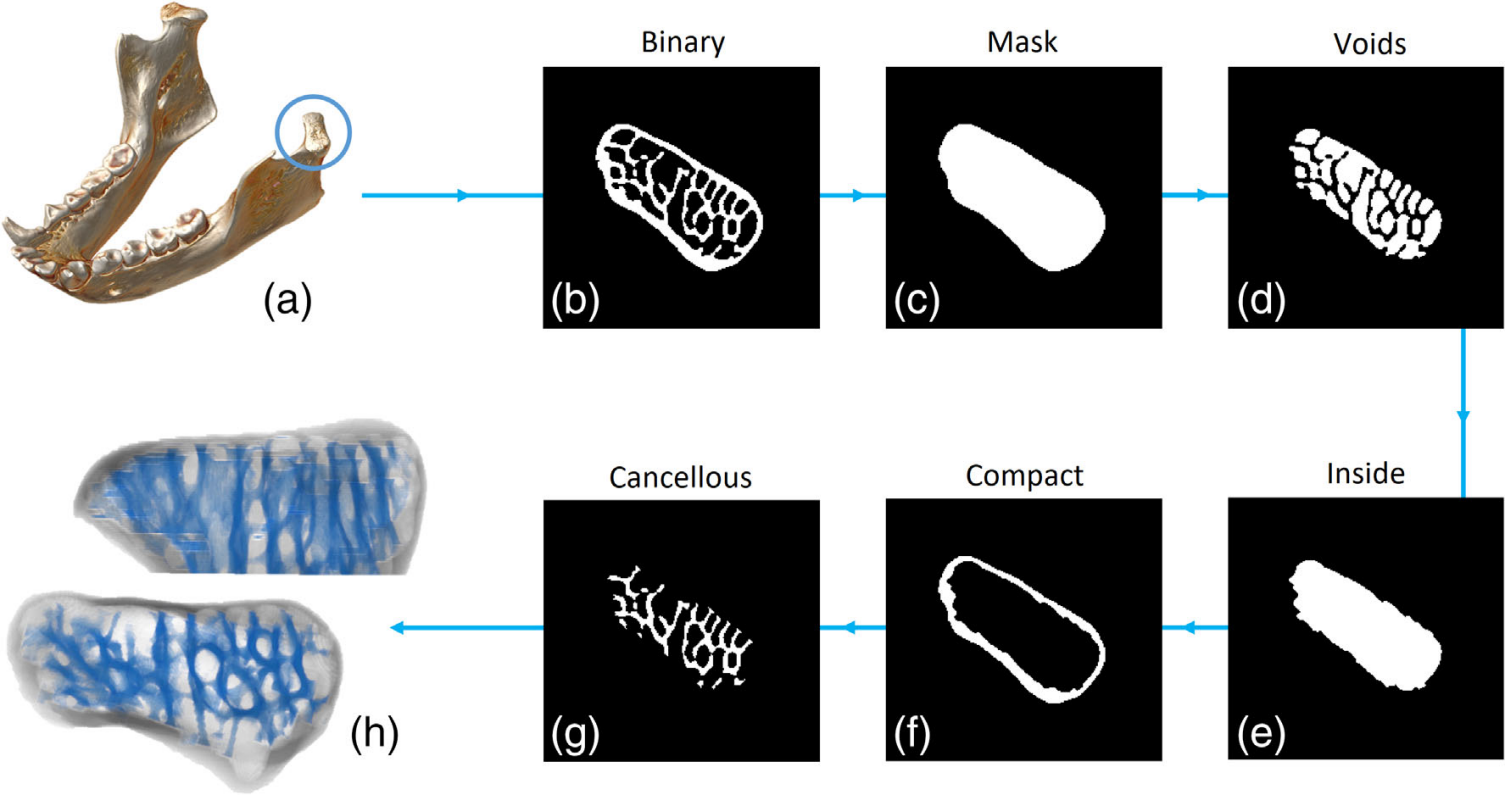
The protocol for the semi‐automatic isolation of cancellous bone shown on the mandibular condyle of *Hylobates lar*. The region of interest (a) is cropped out of the μCT scan and the volume is binarised (b). The binarised image enters the first step of the protocol. Multiple dilations and erosions fill the empty spaces surrounding the cancellous bone, creating a mask (c) of the whole bone region. By subtracting the binary image from the mask (c minus b), the voids are highlighted (d). The voids undergo multiple dilations and erosions, returning the area occupied by voids and cancellous bone (e), which is within the compact bone. By subtracting the inside area from the mask (c minus e), the compact bone is isolated (f). The cancellous bone (g) is then obtained by subtracting the compact bone and the voids from the mask (c minus d minus f). The operation is performed on single μCT slices stacked to obtain a 3D result (h, superior and frontal views of the mandibular condyle)

The protocol consists of five sequential operations alternating dilation/erosion, applied iteratively, to subtractions between images and it is applied sequentially along the *Z* direction of the μCT stack. Figure 2 illustrates the operations for a single 2D image. (Step 1) The white pixels of the binary image (b) undergo multiple dilations that fill the empty spaces within the bone; the same amount of erosions is then applied to shrink the bone back to its original size and external contours. The result is a mask (c) identifying the region occupied by voids, cancellous and compact bone. (Step 2) The subtraction between the pixels of the binary image and the ones of the mask (b–c) provides a new image where only the voids are preserved (d). (Step 3) Multiple dilations of the voids close the spaces occupied by trabecular structures and erosions restore its size and external contours. The white pixels of the resulting image occupy the internal region of the bone (e), the space hosting cancellous bone and voids. (Step 4) The internal region is then subtracted from the mask (c–e), thus isolating the compact bone (f). (Step 5) The cancellous bone (g) is finally obtained by subtracting the voids and the compact bone from the mask (c–d–f). The three‐dimensional (3D) result of the protocol is shown in Figure 2.

The number of iterations for dilation/erosion and the size and shape of the structuring element will depend on the geometric features of the bone to be processed and the resolution of the μCT images. Although the definition of these parameters has to be determined case by case, some rules of thumb exist: the number of iterations increases with increasing image resolution; the number of iterations decreases when increasing the size of the structuring element; the size of the structuring element increases when the image resolution increases; increasing the size of the structuring element increases the level of detail during dilation/erosion. In general, unless the image resolution is very low (e.g., medical CT), a 5 × 5 structuring element is a good choice in most cases and 4 to 6 iterations of dilation/erosion is sufficient to isolate the cancellous bone correctly.

### 2.4 Skeletonization

The result of the isolation protocol is an image stack that is exported as a NIfTI 3D volume using the “oro.nifti” R package (although it could be exported in any format read by Amira/Avizo). The NIfTI format can be then imported inside the Amira/Avizo software, where the skeletonization takes place. Skeletonization returns the minimal geometric descriptor of an image, usually referred to as the “topological skeleton,” by reducing it to a set of connected nodes and branches (Zhou & Toga, 1999) (Figure S2). In the cancellous bone, branches represent the trabeculae while nodes are the points of connecting contiguous trabeculae. Currently, no suitable skeletonization procedure is available in the R environment, while the Amira/Avizo software provides a topological skeleton through the *Auto Skeleton* tool. In the isolated cancellous bone, this tool calculates a distance map of the trabeculae followed by thinning. The topological skeleton is returned as a list of node and branch coordinates, and indices of the connections between them. The skeleton is then exported from Amira/Avizo in ASCII format.

### 2.5 Indices of cancellous complexity

**FIGURE 3:**
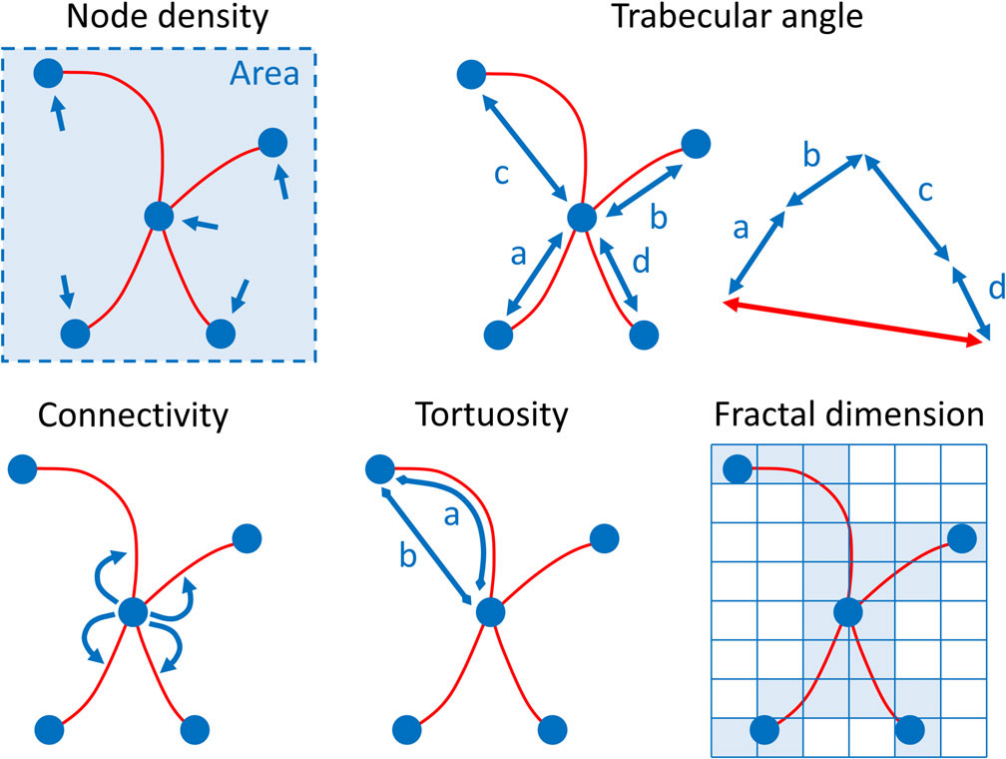
Graphical intuition of the indices measured on the topological skeleton of cancellous bone. For ease of visualization, the indices are shown for a 2D topological skeleton. Node density is represented by the number of nodes per unit area and it is calculated using a kernel density approximation over a discretized space. The trabecular angle (degrees) is measured between a reference axis (not shown) and the unitary resultant (red, double‐headed arrow) of all trabecular directions (blue, double‐headed arrows) obtained by vector sum. Connectivity is the mean number of branches connected to non‐terminal nodes. Tortuosity is the ratio between the arc length of a branch and the linear distance between its starting and ending nodes (a/b). Fractal dimension is an index of complexity measured on the coordinates of the skeleton using the box‐counting algorithm. In this approach, discrete regular grids of decreasing cell size are superimposed over the cancellous skeleton and the number of cells occupied by the skeleton are counted for each grid. Fractal dimension is the slope of the line fitting the number of cells that overlap the skeleton versus the inverse of the cell size

The topological skeleton in ASCII format is read into the R environment using the *readAmiraSkeleton* function in the “indianaBones” R package. The nodes, branches, and connections of the skeleton are processed in R for calculating five indices: node density, trabecular angle, trabecular connectivity, trabecular tortuosity, and fractal dimension. A 2D representation of these measurements is illustrated in Figure 3.

*Node density* is the 3D spatial density of skeleton nodes which is a proxy for trabecular spatial density and the relative proximity of trabecular connections. Node density has been previously used to address bone response to osteoporosis (e.g., Chappard et al., 1988). The link between node density and function is straightforward: higher stress is counteracted by higher density of connections between trabeculae. Here, it is measured using a kernel density approximation (Venables & Ripley, 2002) over a regular 3D grid and it is expressed as number of nodes per cm^3^. The density grid can be plotted in 3D appreciate it visually within individuals. To reduce the effect of size on the calculation of spatial node density, the 3D coordinates of the skeleton nodes can be scaled using a measurement homologous across the dataset. The calculation of node density is performed using the *skelDensity* function in the “indianaBones” R package.

*Trabecular angle* is the 3D angle in degrees between a reference axis and the unitary resultant of all trabecular directions obtained by vector sum in 3D. The idea is that the main direction of trabeculae could detect the trajectory along which the mechanical load is dispersed (Hayes & Snyder, 1981). The direction of single trabeculae is calculated as the difference between the starting and ending nodes of each branch. The reference axis has to be homologous across the sample analyzed and this can be achieved by collecting anatomical or geometric landmarks that define the starting and ending point of the axis. The trabecular angle is calculated using the *skelDirection* function in the “indianaBones” R package. This function also calculates a major axis if a reference axis is not supplied.

*Trabecular connectivity* has been measured via multiple approaches (Ding et al., 2002; Kabel et al., 1999; Odgaard & Gundersen, 1993). Here we define it as the average number of branches connected to each node of the topological skeleton. Only nodes with at least two connections (nonterminal nodes) are considered to calculate the average. Higher connectivity can be expected when cancellous structures are subject to large loads because more connections and more trabeculae allow to spread the load over a wider surface, thus releasing stress on localized areas (Silva & Gibson, 1997). The calculation of trabecular connectivity is performed using the *skelConnectivity* function in the “indianaBones” R package.

*Trabecular tortuosity* has been recognized as a promising indicator of the mechanical behavior of cancellous bone (Roque & Alberich‐Bayarri, 2015). More sinuous, convoluted trabeculae are associated to decreased stiffness (Roque et al., 2012; Roque & Alberich‐Bayarri, 2015). Therefore, tortuosity reflects flexibility when the bone is subject to load. Tortuosity is the ratio between the arc length of a branch and the linear distance between its starting and ending nodes (Roque & Alberich‐Bayarri, 2015). Tortuosity runs from one to infinity, where one corresponds to a straight branch (branch length equals node distance); as the branch gets more convoluted (and its length increases), tortuosity rises and tends to infinity. Infinity represents the absence of a theoretical maximum because the branch has no geometric limit to its length or convolution. Nevertheless, trabeculae are limited to an unknown maximum by the surrounding space and biomechanical requirements, so that infinity is a purely theoretical condition. Because tortuosity is the ratio between two lengths, it is dimensionless. The trabecular tortuosity is calculated using the *skelTortuosity* function in the “indianaBones” R package.

*Fractal dimension* is an index of complexity. It measures the change in detail over different scales of observation (Falconer, 2004). Fractal dimension measured on μCT images or radiographs has been previously applied to the study of cancellous bone (Fazzalari & Parkinson, 1997; Feltrin et al., 2004; Haire et al., 1998; Messent et al., 2005). The rationale is that more complex cancellous structures are more interconnected, which allows spreading the load over a wider surface (Silva & Gibson, 1997). Fractal dimension is here measured on the 3D coordinates of the skeleton branches using the box‐counting algorithm (Annadhason, 2012). In this approach, 3D grids of decreasing cell size (decreasing cell side length, increasing number of cells) are superimposed over the cancellous skeleton. The number of cells overlapping the structure are counted for each subsequent grid: the fractal dimension is the slope of the line fitting the number of cells that overlap the skeleton versus the inverse of the cell size. Fractal dimension is not expressed in units because it measures a fractional dimension (e.g., a fractal dimension of 1.5 is halfway from the one‐dimension of a line and the two dimensions of a square). To reduce size effects, the 3D coordinates of the skeleton branches can be scaled using a measurement homologous across the dataset. The fractal dimension is calculated using the *est.boxcount* function in the “Rdimtools” R package (You, 2020).

### 2.6 Application of the isolation protocol and complexity indices

To show the results of the protocol for cancellous isolation, we use μCT scans of five skeletal regions from five species of primates: the mandibular condyle, the brow ridge, the humerus, the femur and the fibula. Additional details about the specimens are reported in the supplementary information (Table S1). Prior to the application of the protocol, the μCT stacks is binarized by Otsu thresholding using the *otsu* function in the “EBImage” R package. A circular structuring element of 5 × 5 pixel size is used for the dilation/erosion operators. The number of dilation and erosions varied at each step and across bones but never exceeded six.

The usage of complexity indices is shown for a small comparative sample of μCT scanned femoral heads of specimens belonging to seven species of catarrhine primates. Additional details are reported in the supplementary information (Table S2). The aim is to demonstrate the usage, feasibility, and interpretation of the indices in comparative analyses and functional frameworks. Each femoral head is processed using the protocol described above for isolation of cancellous bone and calculation of the indices. Binarization is performed using Otsu thresholding in R (package “EBImage”). The segmented cancellous regions undergo skeletonization using the Amira 5.4.5 software package (FEI Visualization). The indices are then measured in the R environment using the “indianaBones” package. The trabecular angle is calculated with reference to the mediolateral axis, defined as the major axis passing through the femoral neck. Before calculating node density and fractal dimension, the skeleton is scaled on the height of the femoral head. The mediolateral axis and the femoral height are defined in Amira 5.4.5 using the “Landmark” editor. Node density is illustrated using color maps over a 100 × 100 × 100 3D grid used to estimate the kernel density: the colors represent differences in node per cm^3^, increasing from blue to red.

## 3 RESULTS

### 3.1 Cancellous bone isolation

**FIGURE 4:**
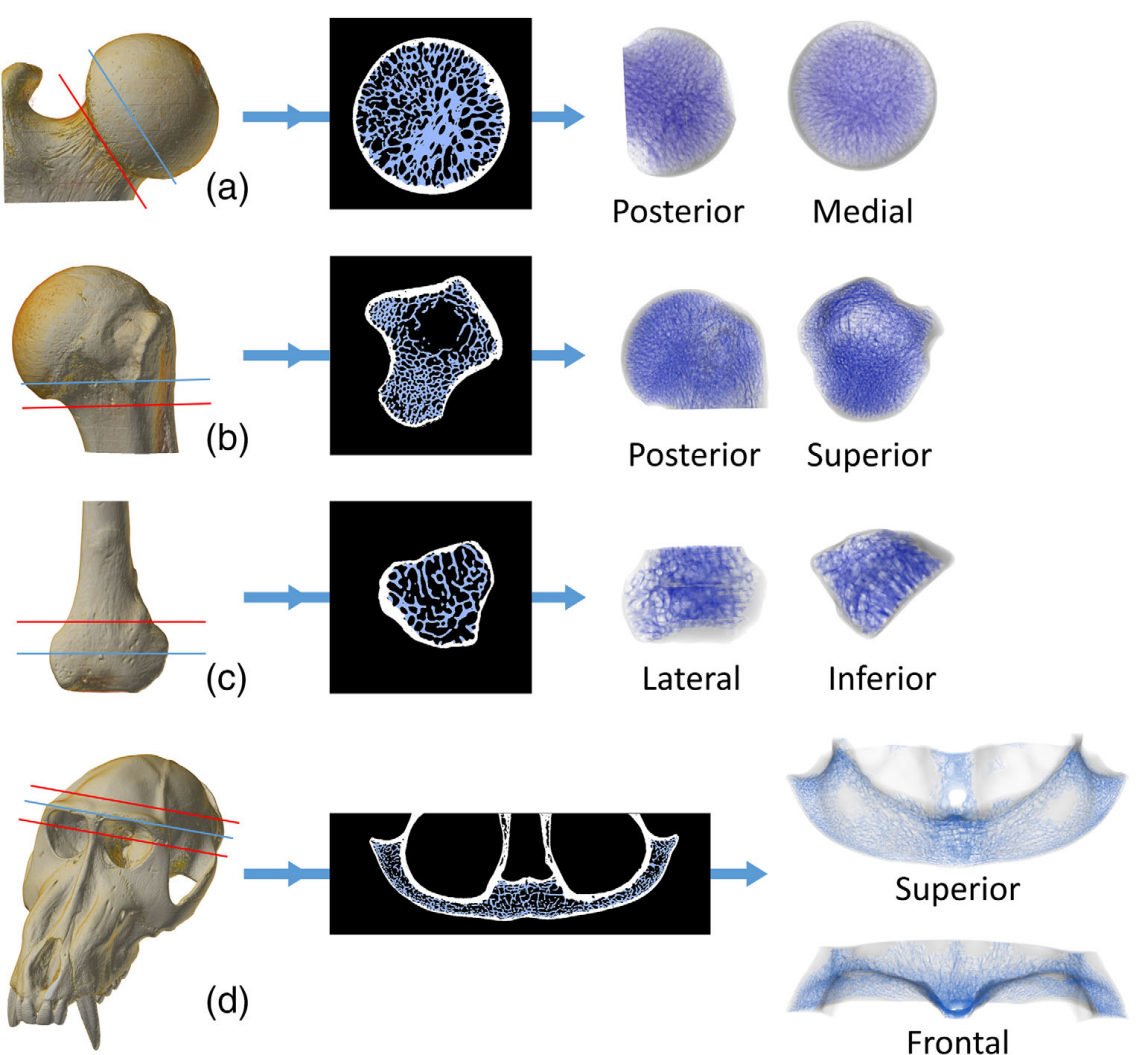
Semi‐automatic isolation of cancellous bone in the femoral head of Symphalangus syndactylus (a), the proximal humerus of *Alouatta caraya* (b), the distal fibula of Cercopithecus albogularis (c) and the brow ridge of Mandrillus sphynx (d). The 3D μCT scan is cut (red line) to limit the cancellous isolation to a region of interest. The results are here shown on a single 2D slice (indicated by the blue line on the 3D scan) and on the full 3D μCT stack (the cutting planes used to isolate the 3D regions of interest is shown in red)

In all the bones tested, the isolation protocol returns the cancellous lattice with, at most, only few small areas of the compact bone left attached (Figure S3). The 2D and 3D results for the mandibular condyle are shown in Figure 2, where this region is used to present the steps of the protocol. For the other skeletal regions, the results are shown in Figure 4.

### 3.2 Application of the complexity indices

**FIGURE 5:**
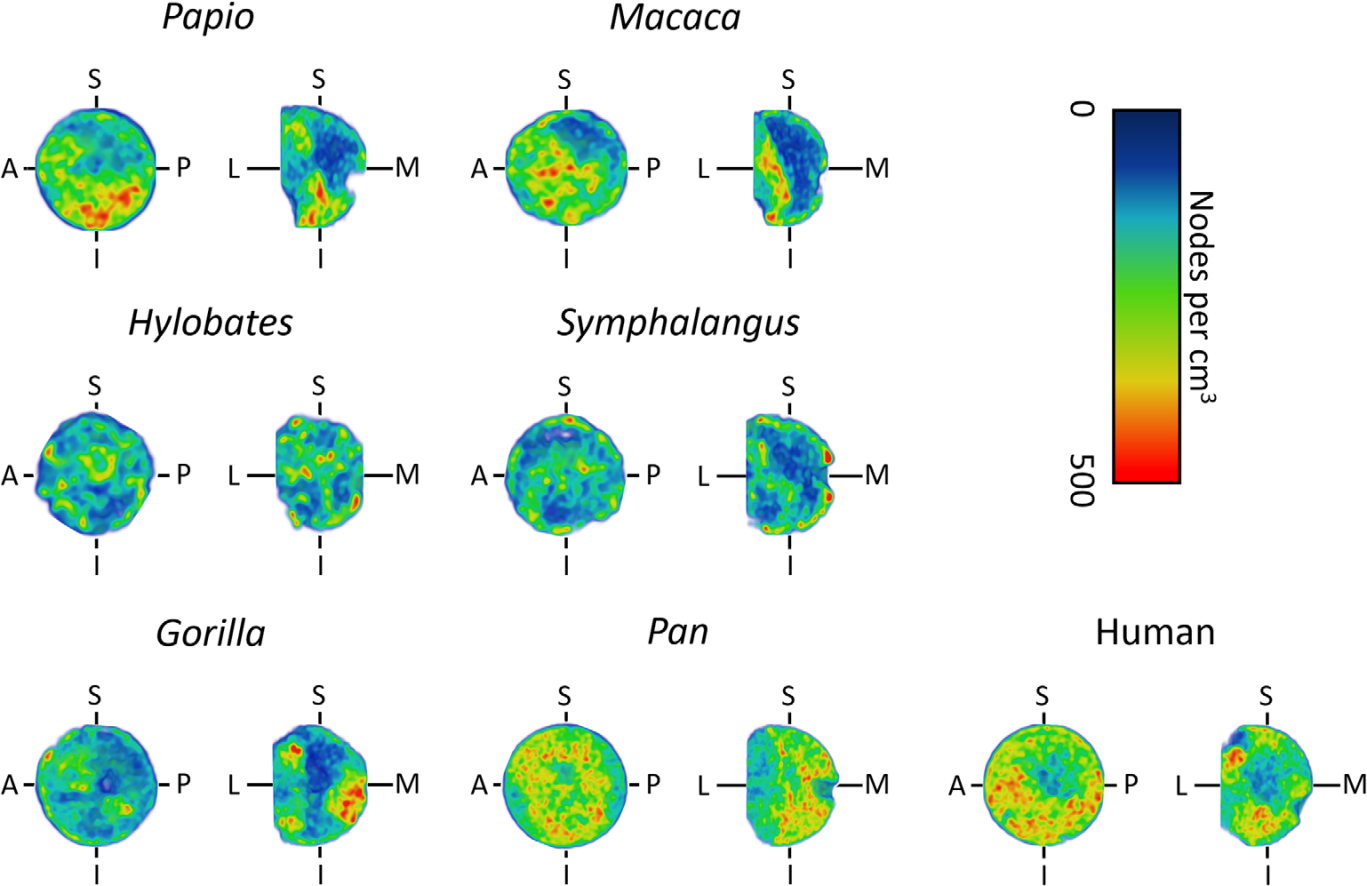
Node density of the femoral head, measured using a kernel density approximation over a regular 3D grid. It is expressed as the number of nodes of the skeletonized cancellous bone per cm^3^. The node density is here shown for a small sample of primates over the coronal (L‐M‐S‐I) and para‐sagittal (A‐P‐S‐I) planes. The density increases from blue to red. (A, anterior; P, posterior; S, superior; I, inferior; L, lateral; M, medial)

Summary statistics of the complexity indices and their *SD* for the specimens analyzed are detailed in Table 1. *Pan*, *Gorilla*, and humans exhibit respective average (47.89 ± 15.84 *SD*, 40.51 ± 16.52 *SD*, and 45.51 ± 14.93 *SD* nodes per cm^3^) and maximum (472.69, 503.97, and 557.71 nodes per cm^3^) density higher than in *Macaca* (mean: 39.38 ± 17.1 *SD*; max: 238.91), *Papio* (mean: 38.81 ± 15.32 *SD*; max: 289.09), *Hylobates* (mean: 37.99 ± 13.12 *SD*; max: 150.73), and *Symphalangus* (mean: 36.93 ± 14.19 *SD*; max: 228.75). The color maps (Figure 5) confirm high node density for humans and *Pan*, localized toward the femoral head's inferior surface in the former and more widespread in the latter. In the coronal view of the human femoral head, the densest areas are localized at the inferior and superolateral sides. In coronal view, *Gorilla* shows the highest node density extending supero‐inferiorly in the lateral aspect of the head, as well as a dense region in the medial aspect at the level of the fovea capitis. In coronal view, *Macaca* and *Papio* show dense regions extending from the superolateral to the inferior aspect of the head and corresponding to the arcuate bundle. The coronal and parasagittal views in *Hylobates* and *Symphalangus* show node densities dispersed across the femoral head than in other species.

**TABLE 1:** Complexity indices calculated on the topological skeleton of the cancellous bone in the femoral head

	Mean node density (nodes/cm^3^)	Max node density (nodes/cm^3^)	*SD*	Trabecular angle (degrees)	Mean tortuosity	*SD*	Mean connectivity	*SD*	Fractal dimension
***Papio***	38.81	289.09	15.32	2.78	1.23	0.25	3.49	0.86	2.39
***Macaca***	39.38	238.91	17.10	5.74	1.11	0.19	3.38	0.78	2.42
***Hylobates***	37.99	150.73	13.12	11.59	1.29	0.36	3.22	0.58	2.3
***Symphalangus***	36.93	228.75	14.19	4.87	1.26	0.31	3.28	0.68	2.37
***Gorilla***	40.51	503.97	16.52	7.43	1.23	0.24	3.53	0.95	2.47
***Pan***	47.89	472.69	15.84	12.21	1.18	0.17	3.81	1.19	2.53
**Human**	45.51	557.71	14.93	4.61	1.21	0.21	3.86	1.29	2.51

*Note*: SD is shown only for the indices for which its calculation was possible. All indices are unitless, except for node density and the trabecular angle. Fractal dimension is here presented as scaled on the height of the femoral head. For the definition and calculation of the indices, see main text.

**FIGURE 6:**
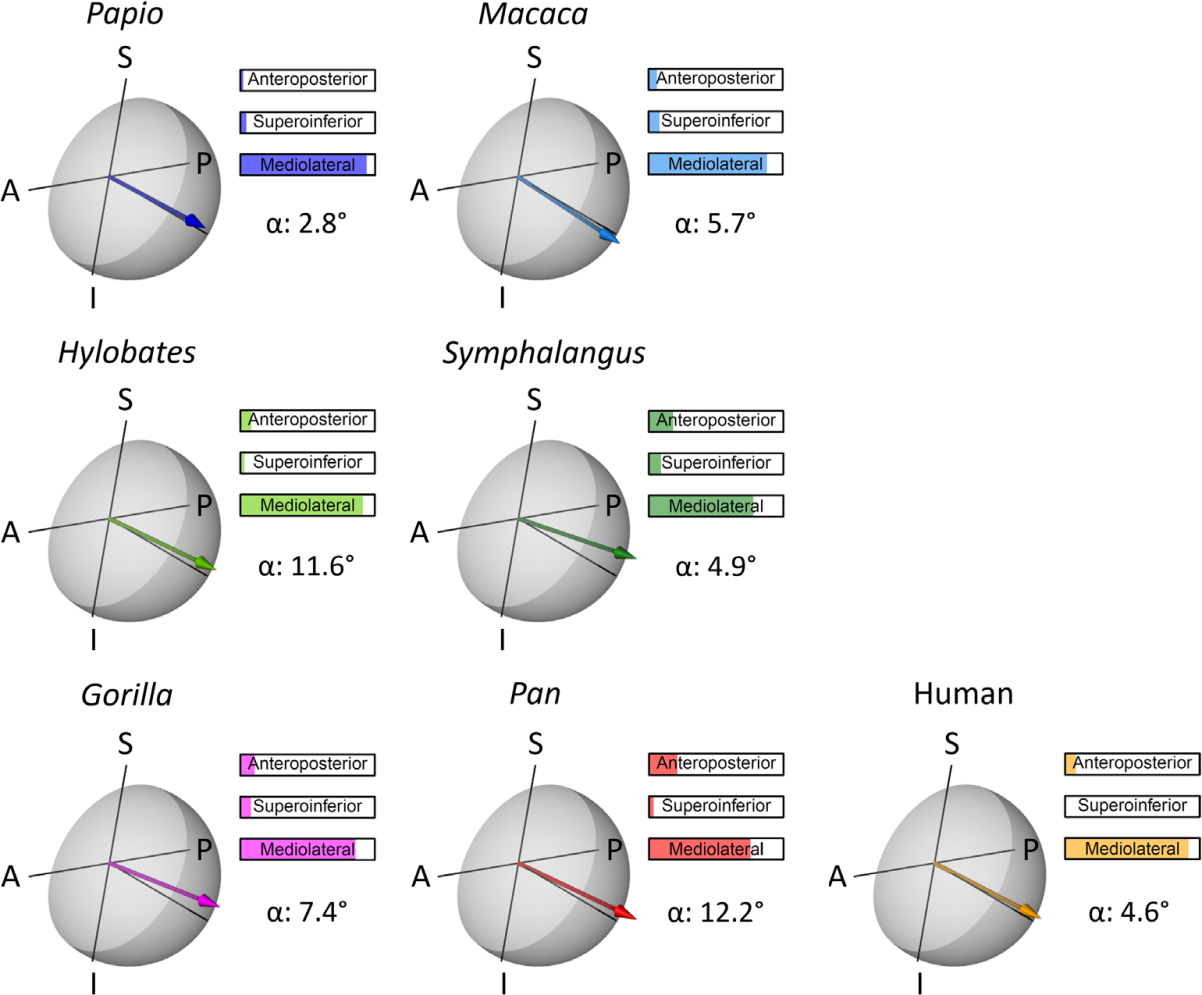
Trabecular angle of the femoral head calculated as the 3D angle between the mediolateral axis and the resultant of all trabecular directions. Trabecular directions are measured on the branches of the skeletonized cancellous bone. The trabecular angle is here shown for a small sample of primates on a transparent model of the femoral head. The mediolateral axis is the line perpendicular to the A‐P‐S‐I plane (para‐sagittal plane). The anteroposterior, supero‐inferior, and mediolateral percentage contributions to the angle are reported. The arrow point is for easing visualization only and does not indicate a verse. (A, anterior; P, posterior; S, superior; I, inferior)

The trabecular angle measured on the femoral head was referenced onto the mediolateral axis. Figure 6 shows the resultant direction of the trabeculae for each specimen and the angles are reported in Table 1. All angles are oriented mediolaterally with only minor departures from the reference axis. Average trabecular connectivity is larger in humans and *Pan* (3.86 ± 1.29 *SD* and 3.81 ± 1.19 *SD*, respectively) than in the other taxa (Table 1). The femoral head of *Gorilla* shows an average of 3.53 ± 0.95 *SD* branches per node, followed by *Papio* (3.49 ± 0.86 *SD*), and *Macaca* (3.38 ± 0.78 *SD*). *Symphalangus* (3.28 ± 0.68 *SD*) and *Hylobates* (3.22 ± 0.58 *SD*) exhibit the lowest average connectivity in the sample. For tortuosity (Table 1), *Macaca* exhibits the lowest average values (1.11 ± 0.19 *SD*). In *Pan* (1.18 ± 0.17 *SD*), humans (1.21 ± 0.21 *SD*), and *Gorilla* (1.23 ± 0.24 *SD*), the average tortuosity is larger than in *Macaca* but comparable to *Papio* (1.23 ± 0.25 *SD*). The highest tortuosity in the sample is displayed by *Hylobates* (1.29 ± 0.36 *SD*) and *Symphalangus* (1.26 ± 0.31 *SD*). Fractal dimension was calculated on the topological skeletons scaled on the height of the femoral head. For *Pan* and humans (Table 1) this index is higher than in all other specimens (2.53 and 2.51 respectively), followed by *Gorilla* (2.47), *Macaca* (2.42), and *Papio* (2.39). *Hylobates* (2.30) and *Symphalangus* (2.37) show the lowest fractal dimensions.

## 4 DISCUSSION

This work presents novel strategies for the processing and analysis of cancellous bone. These approaches do not aim to substitute existing methods but rather to complement them. In addition, these strategies aim to overcome certain limitations of previous studies: the constraints associated to defining homologous cancellous subsamples and the characterization of the inherent complexity of trabecular structures.

The isolation protocol provides a flexible way of separating the cancellous from the compact bone thanks to the sequential application of image processing operators. Figure 4 demonstrates that the protocol can be fruitfully applied to skeletal elements of very different morphology to isolate cancellous bone with precision and avoiding manual segmentation. Also, this tool allows focusing the analysis of cancellous bone on the whole epiphysis rather than on subsamples, thus going past the issues raised in recent literature (Georgiou et al., 2019; Sylvester & Terhune, 2017; Tsegai et al., 2013; Tsegai et al., 2018b) and highlighted in the introduction of this work.

Several tools measure the morphology of cancellous regions directly on the μCT stack (Fajardo & Müller, 2001; Odgaard, 1997). The indices presented in this work rely on the reduction of the cancellous shape to its topological skeleton, which is known to enhance certain geometrical and topological aspects of a shape, such as connectivity, length and direction (Davies, 2004). Therefore, measuring indices of connectivity, tortuosity, density and, overall, complexity on the topological skeleton can be advantageous, because it reduces the effect of features not associated with the complexity of the trabecular lattice (e.g., trabecular thickness and shape). Furthermore, the indices are borrowed from, or inspired by, measurements used in previous literature and we place them within a reproducible framework.

With regard to the functional meaning of the indices, we want to stress that the example presented in this work is meant to illustrate the usage and interpretation of the indices. Clear functional implications cannot be drawn from the results and based on such a small comparative sample. Nevertheless, the results seem to suggest the potential of some of the indices in detecting functional differences: the human, *Pan* and *Gorilla* specimens, whose locomotion produces high mechanical load on the hind limbs, exhibit average node density and connectivity higher than the quadrupedal, and brachiating taxa; the lowest values of fractal dimension are exhibited by *Hylobates* and *Symphalangus*, whose arboreal lifestyle relies consistently on the forelimbs.

Summarizing, the strategy presented in this work innovates the following aspects of cancellous bone studies: (I) it introduces a tool for the isolation of cancellous bone avoiding manual segmentation; (II) it measures indices of complexity taking into account only the topology of the cancellous bone (without confounding, localized cancellous features); (III) provides descriptors of cancellous morphology that can be used to appreciate cancellous complexity numerically (Table 1) and visually (e.g., Figure 5). Further analysis is needed to clarify the extent of functional signal detected by the indices.

## AUTHOR CONTRIBUTIONS

**Alessio Veneziano:** Conceptualization; formal analysis; methodology; project administration; software; supervision; validation; writing‐original draft. **Marine Cazenave:** Conceptualization; data curation; funding acquisition; methodology; resources; software; validation; writing‐original draft; writing‐review & editing. **Fabio Alfieri:** Data curation; formal analysis; methodology; resources; writing‐original draft; writing‐review & editing. **Daniele Panetta:** Data curation; resources; writing‐original draft; writing‐review & editing. **Damiano Marchi:** Conceptualization; project administration; resources; supervision; writing‐original draft; writing‐review & editing.

## Supporting information

**Appendix S1:** Supplementary Information

## Data Availability

Part of the µCT data used in this study is available through the online database Morphosource (https://www.morphosource.org/). Some data is property of the institutions where the specimens were scanned and cannot be made publicly available. Access to this material can be granted by those institutions upon reasonable request. Information about the scans and their identifiers in Morphosource or at their institution are provided in the supplementary information (Table S1 and S2).

## References

[ajpa24272-bib-0001] Andreassen, T. T., & Oxlund, H. (2001). The effects of growth hormone on compact and cancellous bone. Journal of Musculoskeletal and Neuronal Interactions, 2(1), 49–58. 15758476

[ajpa24272-bib-0002] Annadhason, A. (2012). Methods of fractal dimension computation. International Journal of Computer Science and Information Technology & Security., 2(1), 166–169.

[ajpa24272-bib-0003] Bishop, P. J., Clemente, C. J., Hocknull, S. A., Barrett, R. S., & Lloyd, D. G. (2017). The effects of cracks on the quantification of the cancellous bone fabric tensor in fossil and archaeological specimens: A simulation study. Journal of Anatomy, 230(3), 461–470. 10.1111/joa.12569 27896808 PMC5314379

[ajpa24272-bib-0004] Bishop, P. J., Hocknull, S. A., Clemente, C. J., Hutchinson, J. R., Farke, A. A., Barrett, R. S., & Lloyd, D. G. (2018). Cancellous bone and theropod dinosaur locomotion. Part III—Inferring posture and locomotor biomechanics in extinct theropods, and its evolution on the line to birds. PeerJ, 6, e5777. 10.7717/peerj.5777 30402346 PMC6215443

[ajpa24272-bib-0005] Carter, D. R., Orr, T. E., & Fyhrie, D. P. (1989). Relationships between loading history and femoral cancellous bone architecture. Journal of Biomechanics, 22(3), 231–244. 10.1016/0021-9290 2722894

[ajpa24272-bib-0006] Cazenave, M., Oettlé, A., Thackeray, J. F., Nakatsukasa, M., De Beer, F., Hoffman, J., & Macchiarelli, R. (2019). The SKX 1084 hominin patella from Swartkrans member 2, South Africa: An integrated analysis of its outer morphology and inner structure. Comptes Rendus Palevol, 18(2), 223–235.

[ajpa24272-bib-0007] Chappard, D., Alexandre, C., & Riffat, G. (1988). Spatial distribution of trabeculae in iliac bone from 145 osteoporotic females. Acta Anatomica, 132(2), 137–142. 10.1159/000146565 3414359

[ajpa24272-bib-0008] Chen, H., Hayakawa, D., Emura, S., Ozawa, Y., Okumura, T., & Shoumura, S. (2002). Effect of low or high dietary calcium on the morphology of the rat femur. Histology and Histopathology, 17(4), 1129–1136. 10.14670/HH-17.1129 12371141

[ajpa24272-bib-0009] Cooper, C. (1990). Bone mass throughout life: Bone growth and involution. In R. M. Francis & W. C. Dick (Eds.), Osteoporosis (pp. 1–26). Dordrecht: Springer.

[ajpa24272-bib-0010] Core Team, R. (2020). R: A language and environment for statistical computing. Vienna, Austria: R Foundation for Statistical Computing. https://www.R-project.org/

[ajpa24272-bib-0011] Davies, E. R. (2004). Machine vision: Theory. In Algorithms and practicalities (Third ed.). San Francisco, USA: Morgan Kaufmann Publishers.

[ajpa24272-bib-0012] Ding, M., Odgaard, A., Linde, F., & Hvid, I. (2002). Age‐related variations in the microstructure of human tibial cancellous bone. Journal of Orthopaedic Research, 20(3), 615–621. 10.1016/S0736-0266(01)00132-2 12038639

[ajpa24272-bib-0013] Fajardo, R. J., & Müller, R. (2001). Three‐dimensional analysis of nonhuman primate trabecular architecture using micro‐computed tomography. American Journal of Physical Anthropology, 115(4), 327–336. 10.1002/ajpa.1089 11471131

[ajpa24272-bib-0014] Fajardo, R. J., Ryan, T. M., & Kappelman, J. (2002). Assessing the accuracy of high‐resolution X‐ray computed tomography of primate trabecular bone by comparisons with histological sections. American Journal of Physical Anthropology: The Official Publication of the American Association of Physical Anthropologists, 118(1), 1–10. 10.1002/ajpa.10086 11953940

[ajpa24272-bib-0015] Falconer, K. (2004). Fractal geometry: Mathematical foundations and applications (2nd ed.). Chichester: Wiley.

[ajpa24272-bib-0016] Fazzalari, N. L., & Parkinson, I. H. (1997). Fractal properties of subchondral cancellous bone in severe osteoarthritis of the hip. Journal of Bone and Mineral Research, 12(4), 632–640. 10.1359/jbmr.1997.12.4.632 9101375

[ajpa24272-bib-0017] Feltrin, G. P., Stramare, R., Miotto, D., Giacomini, D., & Saccavini, C. (2004). Bone fractal analysis. Current Osteoporosis Reports, 2(2), 53–58. 10.1007/s11914-004-0004-4 16036083

[ajpa24272-bib-0018] Georgiou, L., Kivell, T. L., Pahr, D. H., Buck, L. T., & Skinner, M. M. (2019). Trabecular architecture of the great ape and human femoral head. Journal of Anatomy, 234(5), 679–693. 10.1111/joa.12957 30793309 PMC6481414

[ajpa24272-bib-0019] Goldstein, S. A., Matthews, L. S., Kuhn, J. L., & Hollister, S. J. (1991). Trabecular bone remodeling: An experimental model. Journal of Biomechanics, 24, 135–150. 10.1016/0021-9290(91)90384-Y 1791174

[ajpa24272-bib-0020] Gunnes, M., & Lehmann, E. H. (1995). Dietary calcium, saturated fat, fiber and vitamin C as predictors of forearm compact and trabecular bone mineral density in healthy children and adolescents. Acta Paediatrica, 84(4), 388–392. 10.1111/j.1651-2227.1995.tb13656.x 7795347

[ajpa24272-bib-0021] Gunnes, M., & Lehmann, E. H. (1996). Physical activity and dietary constituents as predictors of forearm compact and trabecular bone gain in healthy children and adolescents: A prospective study. Acta Paediatrica, 85(1), 19–25. 10.1111/j.1651-2227.1996.tb13884.x 8834974

[ajpa24272-bib-0022] Haire, T. J., Hodgskinson, R., Ganney, P. S., & Langton, C. M. (1998). A comparison of porosity, fabric and fractal dimension as predictors of the Young's modulus of equine cancellous bone. Medical Engineering & Physics, 20(8), 588–593. 10.1016/S1350-4533(98)00063-0 9888237

[ajpa24272-bib-0023] Hayes, W. C., & Snyder, B. (1981). Toward a quantitative formulation of Wolff's law in trabecular bone. In S. C. Cowin (Ed.), Mechanical properties of bone. Series AMD (Vol. 45, pp. 43–68). New York: American Society of Mechanical Engineers.

[ajpa24272-bib-0024] Hildebrand, T., Laib, A., Müller, R., Dequeker, J., & Rüegsegger, P. (1999). Direct three‐dimensional morphometric analysis of human cancellous bone: Microstructural data from spine, femur, iliac crest, and calcaneus. Journal of Bone and Mineral Research, 14(7), 1167–1174. 10.1359/jbmr.1999.14.7.1167 10404017

[ajpa24272-bib-0025] Huiskes, R., Ruimerman, R., Van Lenthe, G. H., & Janssen, J. D. (2000). Effects of mechanical forces on maintenance and adaptation of form in trabecular bone. Nature, 405(6787), 704–706. 10.1038/35015116 10864330

[ajpa24272-bib-0026] Kabel, J., Odgaard, A., Van Rietbergen, B., & Huiskes, R. (1999). Connectivity and the elastic properties of cancellous bone. Bone, 24(2), 115–120. 10.1016/S8756-3282(98)00164-1 9951779

[ajpa24272-bib-0027] Kivell, T. L. (2016). A review of trabecular bone functional adaptation: What have we learned from trabecular analyses in extant hominoids and what can we apply to fossils? Journal of Anatomy, 228(4), 569–594. 10.1111/joa.12446 26879841 PMC4804137

[ajpa24272-bib-0028] Kivell, T. L., Skinner, M. M., Lazenby, R., & Hublin, J. J. (2011). Methodological considerations for analyzing trabecular architecture: An example from the primate hand. Journal of Anatomy, 218(2), 209–225. 10.1111/j.1469-7580.2010.01314.x 20977475 PMC3042755

[ajpa24272-bib-0029] Kneissel, M., Boyde, A., Hahn, M., Teschler‐Nicola, M., Kalchhauser, G., & Plenk, H., Jr. (1994). Age‐and sex‐dependent cancellous bone changes in a 4000y BP population. Bone, 15(5), 539–545. 10.1016/8756-3282(94)90278-X 7980965

[ajpa24272-bib-0030] Lazenby, R. A., Skinner, M. M., Kivell, T. L., & Hublin, J. J. (2011). Scaling VOI size in 3D μCT studies of trabecular bone: A test of the over‐sampling hypothesis. American Journal of Physical Anthropology, 144(2), 196–203. 10.1002/ajpa.21385 20979207

[ajpa24272-bib-0031] Little, N., Rogers, B., & Flannery, M. (2011). Bone formation, remodelling and healing. Surgery (Oxford), 29(4), 141–145. 10.1016/j.mpsur.2011.01.002

[ajpa24272-bib-0032] Macchiarelli, R., Bondioli, L., Galichon, V., & Tobias, P. V. (1999). Hip bone trabecular architecture shows uniquely distinctive locomotor behaviour in south African australopithecines. Journal of Human Evolution, 36(2), 211–232. 10.1006/jhev.1998.0267 10068067

[ajpa24272-bib-0033] Messent, E. A., Ward, R. J., Tonkin, C. J., & Buckland‐Wright, C. (2005). Tibial cancellous bone changes in patients with knee osteoarthritis. A short‐term longitudinal study using fractal signature analysis. Osteoarthritis and Cartilage, 13(6), 463–470. 10.1016/j.joca.2005.01.007 15922180

[ajpa24272-bib-0034] Miyakoshi, N. (2004). Effects of parathyroid hormone on cancellous bone mass and structure in osteoporosis. Current Pharmaceutical Design, 10(21), 2615–2627. 10.2174/1381612043383737 15320749

[ajpa24272-bib-0035] Moon, H. S., Won, Y. Y., Kim, K. D., Ruprecht, A., Kim, H. J., Kook, H. K., & Chung, M. K. (2004). The three‐dimensional microstructure of the trabecular bone in the mandible. Surgical and Radiologic Anatomy, 26(6), 466–473. 10.1007/s00276-004-0247-x 15146293

[ajpa24272-bib-0036] Odgaard, A. (1997). Three‐dimensional methods for quantification of cancellous bone architecture. Bone, 20(4), 315–328. 10.1016/S8756-3282(97)00007-0 9108351

[ajpa24272-bib-0037] Odgaard, A., & Gundersen, H. J. G. (1993). Quantification of connectivity in cancellous bone, with special emphasis on 3‐D reconstructions. Bone, 14(2), 173–182. 10.1016/8756-3282(93)90245-6 8334036

[ajpa24272-bib-0038] Otsu, N. (1979). A threshold selection method from gray‐level histograms. IEEE Transactions on Systems, Man, and Cybernetics, 9(1), 62–66. 10.1109/TSMC.1979.4310076

[ajpa24272-bib-0039] Pau, G., Fuchs, F., Sklyar, O., Boutros, M., & Huber, W. (2010). EBImage—An R package for image processing with applications to cellular phenotypes. Bioinformatics, 26(7), 979–981. 10.1093/bioinformatics/btq046 20338898 PMC2844988

[ajpa24272-bib-0040] Pham, D. L., Xu, C., & Prince, J. L. (2000). Current methods in medical image segmentation. Annual Review of Biomedical Engineering, 2(1), 315–337. 10.1146/annurev.bioeng.2.1.315 11701515

[ajpa24272-bib-0041] Profico, A., Buzi, C., Melchionna, M., Piras, P., Raia, P., & Veneziano, A. (2020). Arothron: Geometric Morphometrics analysis and virtual anthropology. R package version 2.0.1.

[ajpa24272-bib-0042] Rafferty, K. L., & Ruff, C. B. (1994). Articular structure and function in Hylobates, Colobus, and Papio. American Journal of Physical Anthropology, 94(3), 395–408. 10.1002/ajpa.1330940308 7943193

[ajpa24272-bib-0043] Räth, C., Monetti, R., Bauer, J., Sidorenko, I., Müller, D., Matsuura, M., Lochmüller, E. M., Zysset, P., & Eckstein, F. (2008). Strength through structure: Visualization and local assessment of the trabecular bone structure. New Journal of Physics, 10(12), 125010. 10.1088/1367-2630/10/12/125010

[ajpa24272-bib-0044] Roque, W. L., & Alberich‐Bayarri, A. (2015). Tortuosity influence on the trabecular bone elasticity and mechanical competence. In R. N. Jorge & J. M. Tavares (Eds.), Developments in medical image processing and computational vision (pp. 173–191). Cham: Springer.

[ajpa24272-bib-0045] Roque, W. L., Arcaro, K., & Lanfredi, R. B. (2012). Trabecular network tortuosity and connectivity of distal radius from microtomographic images. Revista Brasileira de Engenharia Biomédica, 28(2), 116–123. 10.4322/rbeb.2012.017

[ajpa24272-bib-0046] Ryan, T. M., & Ketcham, R. A. (2002). The three‐dimensional structure of trabecular bone in the femoral head of strepsirrhine primates. Journal of Human Evolution, 43(1), 1–26. 10.1006/jhev.2002.0552 12098207

[ajpa24272-bib-0047] Ryan, T. M., & Shaw, C. N. (2012). Unique suites of trabecular bone features characterize locomotor behavior in human and non‐human anthropoid primates. PLoS One, 7(7), e41037. 10.1371/journal.pone.0041037 22815902 PMC3399801

[ajpa24272-bib-0048] Scherf, H., Harvati, K., & Hublin, J. J. (2013). A comparison of proximal humeral cancellous bone of great apes and humans. Journal of Human Evolution, 65(1), 29–38. 10.1016/j.jhevol.2013.03.008 23680068

[ajpa24272-bib-0049] Scherf, H., Wahl, J., Hublin, J. J., & Harvati, K. (2016). Patterns of activity adaptation in humeral trabecular bone in Neolithic humans and present‐day people. American Journal of Physical Anthropology, 159(1), 106–115. 10.1002/ajpa.22835 26293309

[ajpa24272-bib-0050] Serra, J. (1982). Image analysis and mathematical morphology (Vol. 1, 2nd ed.). New York: Academic Press.

[ajpa24272-bib-0051] Silva, M. J., & Gibson, L. J. (1997). Modeling the mechanical behavior of vertebral trabecular bone: Effects of age‐related changes in microstructure. Bone, 21(2), 191–199. 10.1016/S8756-3282(97)00100-2 9267695

[ajpa24272-bib-0052] Sinclair, K. D., Farnsworth, R. W., Pham, T. X., Knight, A. N., Bloebaum, R. D., & Skedros, J. G. (2013). The artiodactyl calcaneus as a potential ‘control bone’. Cautions against simple interpretations of trabecular bone adaptation in the anthropoid femoral neck. Journal of Human Evolution, 64(5), 366–379. 10.1016/j.jhevol.2013.01.003 23481347

[ajpa24272-bib-0053] Sylvester, A. D., & Terhune, C. E. (2017). Trabecular mapping: Leveraging geometric morphometrics for analyses of trabecular structure. American Journal of Physical Anthropology, 163(3), 553–569. 10.1002/ajpa.23231 28432829

[ajpa24272-bib-0054] Tsegai, Z. J., Kivell, T. L., Gross, T., Nguyen, N. H., Pahr, D. H., Smaers, J. B., & Skinner, M. M. (2013). Trabecular bone structure correlates with hand posture and use in hominoids. PLoS One, 8(11), e78781. 10.1371/journal.pone.0078781 24244359 PMC3828321

[ajpa24272-bib-0055] Tsegai, Z. J., Skinner, M. M., Pahr, D. H., Hublin, J. J., & Kivell, T. L. (2018a). Ontogeny and variability of trabecular bone in the chimpanzee humerus, femur and tibia. American Journal of Physical Anthropology, 167(4), 713–736. 10.1002/ajpa.23696 30159927

[ajpa24272-bib-0056] Tsegai, Z. J., Skinner, M. M., Pahr, D. H., Hublin, J. J., & Kivell, T. L. (2018b). Systemic patterns of trabecular bone across the human and chimpanzee skeleton. Journal of Anatomy, 232(4), 641–656. 10.1111/joa.12776 29344941 PMC5835784

[ajpa24272-bib-0057] Tu, S. J., Wang, S. P., Cheng, F. C., Weng, C. E., Huang, W. T., Chang, W. J., & Chen, Y. J. (2017). Attenuating trabecular morphology associated with low magnesium diet evaluated using micro computed tomography. PLoS One, 12(4), e0174806. 10.1371/journal.pone.0174806 28369124 PMC5378393

[ajpa24272-bib-0058] Urbach, E. R., & Wilkinson, M. H. (2007). Efficient 2‐D grayscale morphological transformations with arbitrary flat structuring elements. IEEE Transactions on Image Processing, 17(1), 1–8. 10.1109/tip.2007.912582 18229799

[ajpa24272-bib-0059] Urbanek, S. (2020). TIFF: Read and write TIFF images. R package version 0.1‐7. https://CRAN.R-project.org/package=tiff

[ajpa24272-bib-0060] Vala, H. J., & Baxi, A. (2013). A review on Otsu image segmentation algorithm. International Journal of Advanced Research in Computer Engineering & Technology, 2(2), 387–389. 10.14445/22312803/IJCTT-V33P117

[ajpa24272-bib-0061] Venables, W. N., & Ripley, B. D. (2002). Modern applied statistics with S (4th ed.). Cham: Springer.

[ajpa24272-bib-0062] Villa, C., Hansen, M. N., Buckberry, J., Cattaneo, C., & Lynnerup, N. (2013). Forensic age estimation based on the trabecular bone changes of the pelvic bone using post‐mortem CT. Forensic Science International, 233(1–3), 393–402. 10.1016/j.forsciint.2013.10.020 24314546

[ajpa24272-bib-0063] Whitcher, B., Schmid, V. J., & Thornton, A. (2011). Working with the DICOM and NIfTI data standards in R. Journal of Statistical Software, 44(6), 1–28. http://www.jstatsoft.org/v44/i06/

[ajpa24272-bib-0064] You, K. (2020). Rdimtools: An R package for Dimension Reduction and Intrinsic Dimension Estimation. *arXiv preprint* arXiv:2005.11107.

[ajpa24272-bib-0065] Zhou, Y., & Toga, A. W. (1999). Efficient skeletonization of volumetric objects. IEEE Transactions on Visualization and Computer Graphics, 5(3), 196–209. 10.1109/2945.795212 20835302 PMC2936771

