# Supplementary material for: Novel strategies for the characterization of cancellous bone morphology: Virtual isolation and analysis
Source: Am J Phys Anthropol. 2021 Apr 3;175(4):920–30. doi: 10.1002/ajpa.24272 (PMC8359981; doi:10.1002/ajpa.24272)
Supplement: Supplementary file 1 — Appendix S1: Supplementary Information [file AJPA-175-920-s001.docx]

**Novel strategies for the characterisation of cancellous bone morphology: virtual isolation and analysis – Supplementary Information**

Alessio Veneziano^1^, Marine Cazenave^2,3^, Fabio Alfieri^4,5^, Daniele Panetta^6^, Damiano Marchi^7,8^

1 Synchrotron Radiation for Medical Physics (SYRMEP), Elettra-Sincrotrone Trieste S.C.p.A., Basovizza, Trieste, Italy

2 Skeletal Biology Research Centre at the School of Anthropology and Conservation, University of Kent, Canterbury, UK

3 Department of Anatomy and Histology, Sefako Makgatho Health Sciences University, Pretoria, South Africa

4 Institut für Biologie, Humboldt Universität zu Berlin, Berlin, Germany.

5 Museum für Naturkunde, Leibniz-Institut für Evolutions- und Biodiversitätsforschung, Berlin, Germany

6 Istituto di Fisiologia Clinica, Consiglio Nazionale delle Ricerche (CNR), Pisa, Italy

7 Department of Biology, Università di Pisa, Pisa, Italy

8 Evolutionary Studies Institute and Centre for Excellence in PalaeoSciences, University of the Witwatersrand, Johannesburg, South Africa

Corresponding Author: Alessio Veneziano


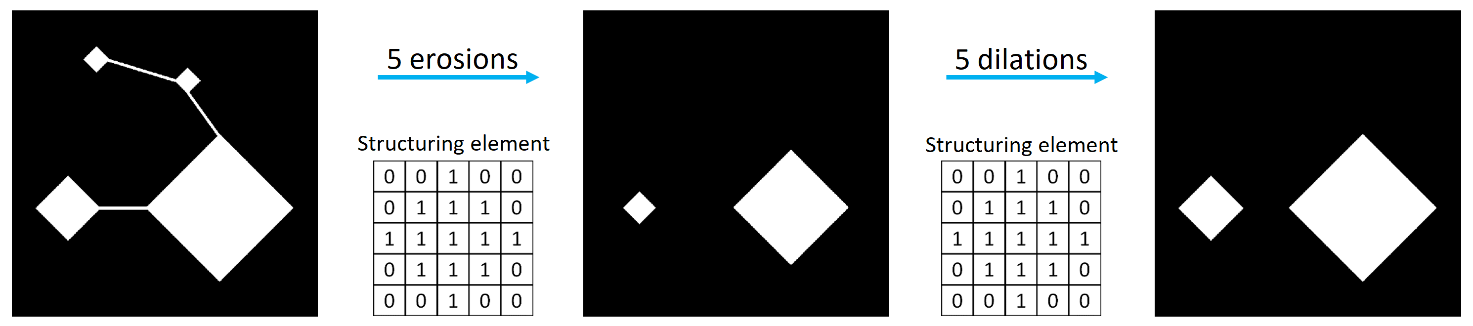


**Figure S1** Action and irreversibility of Erosion and Dilation operators. The image (left) is eroded using a 5x5 diamond-shaped structuring element. The structuring element provides the operators with the transformation pattern. Dilation and erosion do not produce opposite results as they are not complementary operations. The effect of repeated dilations determines the closure of holes or flattening of concavities within an image. When such structures are lost or heavily attenuated, erosions do not restore the original appearance of the image. Similarly, when sequential erosions are applied, small components can be lost from the image and dilation does not bring them back. This situation is depicted above. After five erosions, the smaller and thinner components of the image are lost, while the large parts shrink in size (centre). Five following dilations restore the size of the large components but the small part cannot be restored (right). The irreversibility of dilation and erosion is the core of the protocol here presented as it allows losing the connections between compact and trabecular regions.


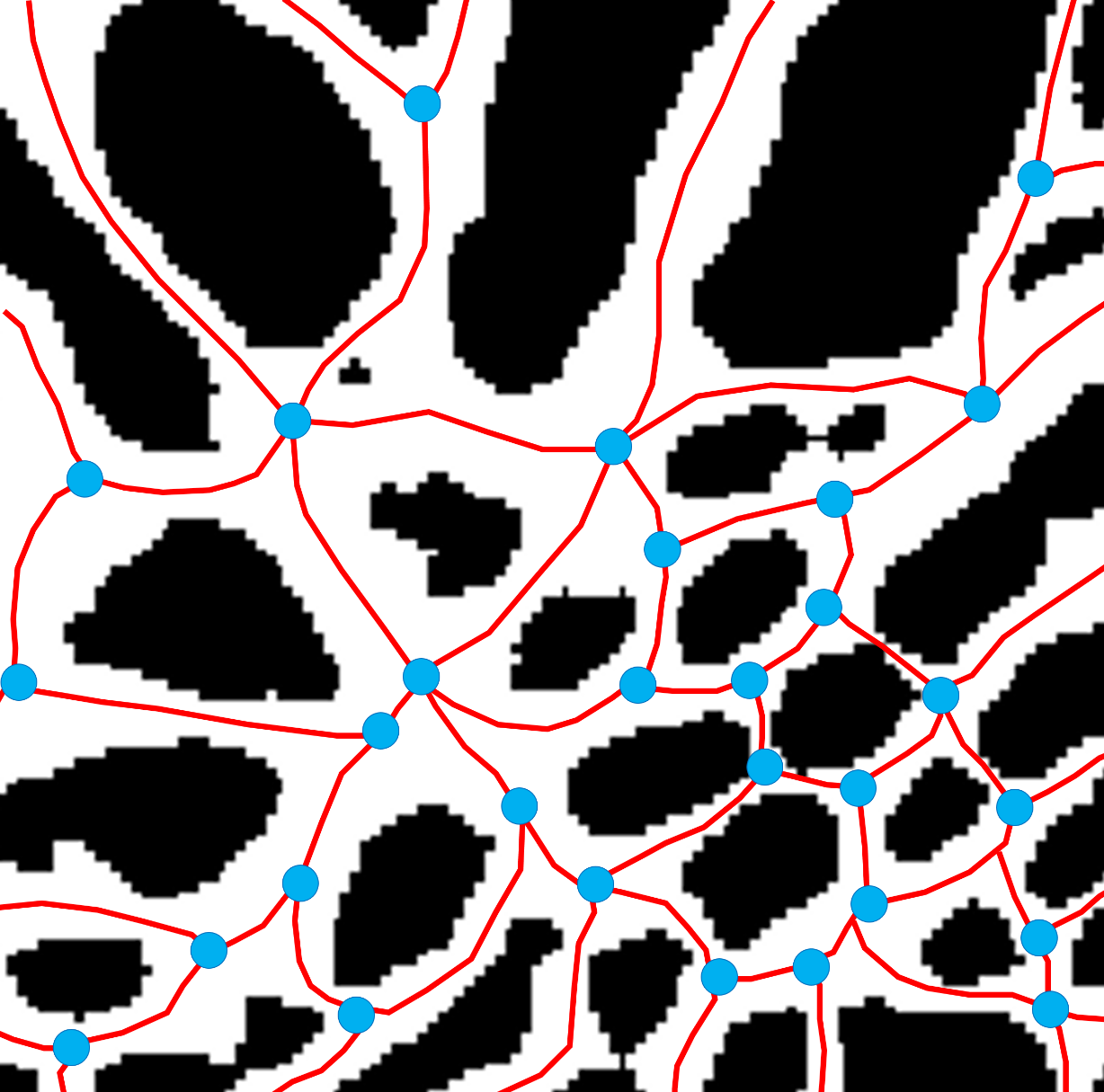


**Figure S2** Skeletonisation of the cancellous bone. The cancellous architecture can be reduced to its minimal morphology by thinning the cancellous bone. The result is a lattice structure consisting of branches (red) and nodes (blue). The topological and geometric features of the skeleton can be used to measure proxies of its complexity. For the sake of visualisation, the skeletonisation is here shown on a 2D image, while for the methods presented in this paper it is performed in 3D.


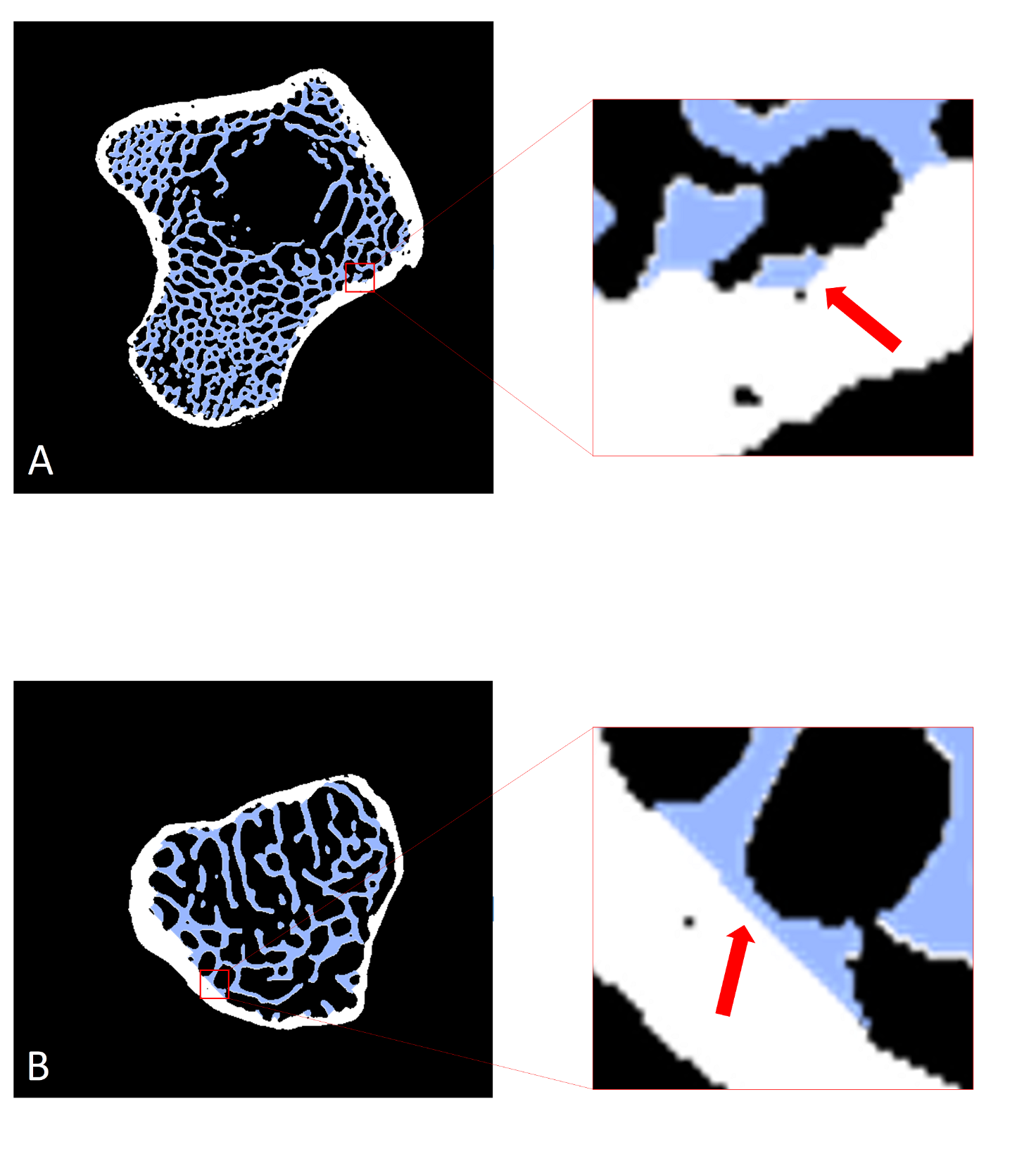


**Figure S3** Details of errors that can be encountered using the isolation protocol presented in this paper. A) Detail of the isolated cancellous bone of the proximal humerus of Alouatta caraya. Small parts or irregularities of the internal side of the cortical bone (red arrow) can be included in the cancellous selection, in particular when close to small holes within the cortical bone. B) Detail of the isolated cancellous bone of the distal fibula of Cercopithecus albogularis. Bridges (red arrow) between contiguous cancellous element can be connected when the element touches the cortical bone directly. Some cortical bone can therefore be retained in the selection. These errors are usually localised to small areas and their effect on the overall calculation of cancellous statistics can be considered negligible.

**Table S1** Details of the specimens (μCT) used to test the protocol of cancellous bone isolation.

| **Species** | **Skeletal region** | **Side** | **Isotropic voxel size** | **Source** | **Scanning facility** | **Data available at** |
| --- | --- | --- | --- | --- | --- | --- |
| *Hylobates lar* | Mandibular condyle | Left | 0.067 mm^3^ | The Museum of Comparative Zoology at Harvard University (USA) | Microfocus X-ray tomography facility, Center for Nanoscale Systems (CNS), Harvard University (USA) | Morphosource  ID: MCZ:Mamm:41421 |
| *Mandrillus sphynx* | Brow ridge | - | 0.084 mm^3^ | The American Museum of Natural History (USA) | Microscopy and Imaging Facility (MIF), The American Museum of Natural History (USA) | Morphosource  ID: amnh:Mamm:m-89362 |
| *Alouatta caraya* | Proximal humerus | Right | 0.037 mm^3^ | The American Museum of Natural History (USA) | Center for Quantitative Imaging (CQI) of the Penn State University (USA) | Morphosource  ID: amnh: Mamm:m-211502 |
| *Symphalangus syndactylus* | Femoral head | Right | 0.037 mm^3^ | The Smithsonian National Museum of Natural History (USA, courtesy of T. M. Ryan) | Microfocus X-ray tomography facility, the Smithsonian National Museum of Natural History (USA) | Marine Cazenave  ID: NMNH143579 |
| *Cercopithecus albogularis* | Distal fibula | Right | 0.023 mm^3^ | “La Specula” Natural History Museum (Italy) | Institute of Clinical Physiology, the National Research Council (CNR-IFC), Pisa (Italy) | Daniele Panetta  ID: XALT-FIB-3011 |

**Table S2** Details of the femoral head sample (μCT) used to show the application of the complexity indices measured on the topological skeleton.

| **Species** | **Side** | **Isotropic voxel size** | **Source** | **Scanning facility** | **Data available at** |
| --- | --- | --- | --- | --- | --- |
| *Homo sapiens* | Right | 0.064 mm^3^ | The Pretoria Bone Collection, Department of Anatomy of the University of Pretoria (South Africa) | Microfocus X-ray tomography facility (MIXRAD), the South African Nuclear Energy Corporation SOC Ltd (Necsa, South Africa) | Marine Cazenave  ID: PBCX1699 |
| *Pan troglodytes* | Left | 0.041 mm^3^ | The Evolutionary Studies Institute, University of Witwatersrand (South Africa) | The Microfocus X-ray tomography facility, the Evolutionary Studies Institute (ESI), University of Witwatersrand (South Africa) | Marine Cazenave  ID: Za1355 |
| *Gorilla gorilla* | Right | 0.072 mm^3^ | The R.A. Dart skeletal collection, University of Witwatersrand (South Africa) | The Microfocus X-ray tomography facility, the Evolutionary Studies Institute (ESI), University of Witwatersrand (South Africa) | Marine Cazenave  ID: Za1312 |
| *Hylobates lar* | Right | 0.053 mm^3^ | The Museum of Comparative Zoology at Harvard University (USA, courtesy of M. Pina) | Microfocus X-ray tomography facility, Center for Nanoscale Systems (CNS), Harvard University (USA) | Marine Cazenave  ID: MCZ41412 |
| *Symphalangus syndactylus* | Right | 0.037 mm^3^ | The Smithsonian National Museum of Natural History (USA, courtesy of T. M. Ryan) | Microfocus X-ray tomography facility, the Smithsonian National Museum of Natural History (USA) | Marine Cazenave  ID: NMNH143579 |
| *Papio ursinus* | Right | 0.066 mm^3^ | The Department of Anatomy and Histology, the Sefako Makgatho Health Sciences University (South Africa) | Microfocus X-ray tomography facility (MIXRAD), the South African Nuclear Energy Corporation SOC Ltd (Necsa, South Africa) | Marine Cazenave  003 |
| *Macaca fuscata* | Right | 0.046 mm^3^ | The Primate Research Institute of Kyoto University (Japan, courtesy of R. Macchiarelli) | X-ray synchrotron radiation micro-tomography (SR-μXCT), beamline ID 17 of the European Synchrotron Radiation Facility (ESRF, France) | Marine Cazenave  ID: KAS276 |

**Example code for segmentation of cancellous bone and calculation of the complexity indices.**

################################################################Install packages

if (!requireNamespace("BiocManager",quietly=T))

install.packages("BiocManager")

BiocManager::install("EBImage")

install.packages("mmand",dependencies=T)

install.packages("Rdimtools",dependencies=T)

install.packages("devtools",dependencies=T)

devtools::install_github("https://github.com/AlessioVeneziano/IndianaBones",local=F)

##################################################################Call packages

library(EBImage)

library(mmand)

library(Rdimtools)

library(indianaBones)

###################################################Isolation of the cancellous bone

data(exampleStack)

strel<-makeBrush(5,"disc")

seg<-splitBone(Stack,strel,3,2,3,0)

image(Stack[,,30],col=c(1,0))

image(seg$comp[,,30],col=c(1,0))

image(seg$trab[,,30],col=c(1,0))

################################################Calculation of the complexity indices

data(exampleSkeleton)

dens<-skelDensity(skel,"node",F)

conn<-skelConnectivity(skel)

len<-skelLength(skel)

thi<-skelThickness(skel)

tor<-skelTortuosity(skel)

ang<-skelDirection(skel,1)

fd<-est.boxcount(skel[[5]],50)$estdim

######################################END OF SCRIPT
